# Evaluation of single-fraction high dose FLASH radiotherapy in a cohort of canine oral cancer patients

**DOI:** 10.3389/fonc.2023.1256760

**Published:** 2023-09-11

**Authors:** Betina Børresen, Maja L. Arendt, Elise Konradsson, Kristine Bastholm Jensen, Sven ÅJ. Bäck, Per Munck af Rosenschöld, Crister Ceberg, Kristoffer Petersson

**Affiliations:** ^1^ Department of Veterinary Clinical Sciences, University of Copenhagen, Frederiksberg, Denmark; ^2^ Medical Radiation Physics, Department of Clinical Sciences, Lund University, Lund, Sweden; ^3^ Veterinärhuset Öresund, Limhamn, Sweden; ^4^ Radiation Physics, Department of Hematology, Oncology and Radiation Physics, Skåne University Hospital, Lund, Sweden; ^5^ Department of Oncology, Oxford Institute for Radiation Oncology, University of Oxford, Oxford, United Kingdom

**Keywords:** radiotherapy, FLASH radiotherapy, osteoradionecrosis, late toxicity, canine cancer, translational research, ultra-high dose rate, veterinary trial

## Abstract

**Background:**

FLASH radiotherapy (RT) is a novel method for delivering ionizing radiation, which has been shown in preclinical studies to have a normal tissue sparing effect and to maintain anticancer efficacy as compared to conventional RT. Treatment of head and neck tumors with conventional RT is commonly associated with severe toxicity, hence the normal tissue sparing effect of FLASH RT potentially makes it especially advantageous for treating oral tumors. In this work, the objective was to study the adverse effects of dogs with spontaneous oral tumors treated with FLASH RT.

**Methods:**

Privately-owned dogs with macroscopic malignant tumors of the oral cavity were treated with a single fraction of ≥30Gy electron FLASH RT and subsequently followed for 12 months. A modified conventional linear accelerator was used to deliver the FLASH RT.

**Results:**

Eleven dogs were enrolled in this prospective study. High grade adverse effects were common, especially if bone was included in the treatment field. Four out of six dogs, who had bone in their treatment field and lived at least 5 months after RT, developed osteoradionecrosis at 3-12 months post treatment. The treatment was overall effective with 8/11 complete clinical responses and 3/11 partial responses.

**Conclusion:**

This study shows that single-fraction high dose FLASH RT was generally effective in this mixed group of malignant oral tumors, but the risk of osteoradionecrosis is a serious clinical concern. It is possible that the risk of osteonecrosis can be mitigated through fractionation and improved dose conformity, which needs to be addressed before moving forward with clinical trials in human cancer patients.

## Introduction

FLASH radiotherapy (RT) is a novel cancer treatment modality, which features ultra-high dose rate RT delivered at ≥30-40 Gy/second (1). The first FLASH RT studies performed in experimental cancer models and veterinary cancer patients have all been published within the last few years (2–5). These suggest that FLASH RT diminishes normal tissue toxicity compared to conventional RT without losing anti-tumor efficacy, which has been named the “FLASH effect” (6). This has made FLASH RT a growing research topic world-wide, with the promising potential to obtain a new tool for differential sparing of normal tissue, complementary to fractionation and target dose conformity. Accordingly, multiple centers world-wide are currently either planning or recruiting for their first early-phase human FLASH RT trials, despite the lack of studies describing long-term effects. A report from the first human FLASH RT clinical trial (the FAST-01 trial) was recently published, demonstrating feasibility of treating human patients with painful bone metastases (7).

In 2021, we published our initial experiences of treating 10 canine cancer patients with electron FLASH on a modified clinical linear accelerator, demonstrating that FLASH RT was safe and effective in the early setting post treatment (3). However, since then, a Swiss study has shown severe adverse effects after FLASH RT in feline cancer patients 9-15 months post treatment (5), clearly demonstrating the need for longer follow up times when reporting on the safety of FLASH RT for clinical patients.

It is evident that there is still much to learn, especially about the risk of late adverse effects induced by FLASH RT, before it can be safely implemented for use in non-terminal human cancer patients. Consequently, we and other groups have focused on canine and feline cancer patients to explore the potential of FLASH RT in a clinically relevant setting (2–5). Importantly, canine cancer patients develop cancer spontaneously in an environment shared with humans and with tumor heterogeneity and -biology similar to humans, as opposed to experimental animal models (8–10). The clinical course in canine cancer patients is similar to that of humans including treatment response, resistance, recurrence, metastasis and death, however the time course is generally accelerated.

The present study focuses on FLASH RT for oral malignant tumors. Treating tumors of the oral cavity with conventional fractionated RT is commonly associated with severe adverse effects to the oral and perioral tissues, which may severely impact the quality of life for both human and veterinary cancer patients during and after RT (11, 12). It is currently unknown if the previously demonstrated normal tissue sparing effects of FLASH RT will also include the very sensitive oral tissues, in which case FLASH RT could be especially advantageous for the many human head and neck cancer patients treated with RT. Consequently, the overall aim of this study was to evaluate the safety of FLASH RT for treating oral tumors, with efficacy as a secondary aim. In order to investigate this, a clinical study was performed in canine cancer patients with spontaneous malignant oral tumors. The dogs were treated with a single fraction of high dose FLASH RT and subsequently followed for 12 months. Our hypothesis was that FLASH RT would be safe with only minimal adverse effects to the oral cavity.

## Materials and methods

### Study design

The study was designed as a single-armed interventional study with the main aim of showing feasibility and safety of using single-fraction high dose FLASH RT to treat malignant tumors of the oral cavity. The clinical efficacy of FLASH RT was a secondary aim. The study was not designed to provide statistical comparisons with other treatment modalities or protocols.

### Canine patient population

Dogs presenting to the oncology clinic at the University Hospital for Companion Animals (University of Copenhagen, Denmark) or to a private veterinary hospital (Veterinärhuset Öresund, Limhamn, Sweden) were prospectively included if they had a macroscopic malignant tumor located in the oral cavity that was either inoperable or where the owners had decided against standard therapy. The diagnosis had to be confirmed by histopathology for all other tumor types than mast cell tumors, for which cytology was considered adequate. The dogs were allowed to receive other non-surgical treatments concurrently. Dogs, who were poor candidates for anesthesia, such as those with severe heart-, liver- or kidney disease, were excluded from the study.

Criteria for discontinuing patient inclusion were development of Veterinary Radiation Oncology Group (VRTOG) grade 3 late adverse effects to the oral mucosa, skin or bone. VRTOG grade 3 acute adverse effects including minor ulcerations that would subsequently heal were allowed.

### Ethics

Prior to study inclusion, the dogs’ owners were orally informed about the potential risks related to the study and following this, they were asked to sign a consent form. The study was approved by the Local Ethical and Administrative Committee at Department of Veterinary Clinical Sciences, University of Copenhagen, the Danish Experimental Animals Inspectorate (2020–15–0201–00429), the Swedish Board of Agriculture (5.2.18-02830/2020), and the Animal Experiments Committee in Lund, Malmö (5.8.18-14316/2021).

### FLASH treatments and dosimetry

FLASH RT was delivered on a clinical Elekta Precise linear accelerator with Integrity software version 1.2 (Elekta AB, Stockholm, Sweden). The accelerator was temporarily modified to be controlled on a pulse-by-pulse basis and to deliver 10 MeV electron FLASH irradiation with a nominal pulse repetition frequency of 200 Hz and pulse width of 3.5 µs (13). The dose-per-pulse (DPP) ranged between 1.3 Gy and 2.3 Gy, resulting in average dose rates ≥115 Gy/s, pulse dose rates ≥3.5*10^5^ Gy/s, and treatment times ≤305 ms. Patients were treated at a source-to-surface distance (SSD) of 70 cm using an established setup previously described (3). The beam characteristics in terms of percentage depth dose curves and lateral dose profiles have been previously reported by Konradsson et al. (3). Pre-treatment dosimetry was performed with GafChromic EBT-XD film (Ashland Specialty Ingredients G.P., Bridgewater, New Jersey, USA) in phantoms mimicking the treatment geometry to determine the DPP and the number of pulses for each patient. During treatment, *in vivo* dose measurements were performed using EBT-XD film at the skin surface in the center of the beam to verify the delivered dose. The EBT-XD film batch was calibrated in a clinical 10 MeV beam, against an ion-chamber traceable to a standard laboratory for a dose range of 1-40 Gy. In two cases (dogs no 5 and 11), the established setup with an SSD of 70 cm, with a 5 cm gap between the patient and the distal end of the collimator, was not feasible due to the position of the tumors in the palate. Consequently, the treatments were delivered using cylindrical PMMA applicators aligned perpendicular to the collimator using soft docking (3). In these cases, films were placed in the collimator block cut-out instead of at the patient surface, and subsequently related to the dose delivered at the dose maximum via the pre-treatment phantom measurements. A picture of the setup with and without the PMMA applicators can be seen in **Supplementary Figure 1**.

In some dogs, lead shields were used as beam stoppers to protect normal tissue, and tissue equivalent bolus material (Elasto-Gel EP Padding, Southwest Technologies, North Kansas City, Missouri, USA) was used to reduce the treatment depth and increase the surface dose.

The treatments were delivered in a single fraction with prescribed doses at the depth of dose maximum between 30 and 42 Gy. The prescribed dose was based on the findings from our previous phase I dose-escalation study (3), the findings from the original cat dose-escalation study by Vozenin and colleagues (2), and individualized to each patient, within the given range, depending on the expected radiosensitivity of the tumor type, the normal tissues included and when in the inclusion period they were treated. Dogs that were treated in the beginning of the project (dog no 4, 9 and 10) were treated with 30 Gy, since this dose had been found to be safe in previous studies (2, 3). Following this, all dogs were treated with 35 Gy or more depending on tumor and patient characteristics. For example, dog no 3 with an oral malignant melanoma was treated with 40 Gy since this tumor type has a low alpha/beta-ratio, and the tumor was very extensive, so the dog needed effective treatment immediately.

The dogs were sedated for the treatments with dexmedetomidine (2-4mcg/kg) and butorphanol (0.2-0.3mg/kg) IV. In some cases, propofol (1-4mg/kg) was added. In most cases, oxygen was supplied via a face mask, but one dog (dog no 11) with a large oropharyngeal tumor partially obstructing the airways was intubated with an endotracheal tube.

### Assessment of adverse effects and efficacy

Control visits were planned at 7 days, 1 month, 3 months, 6 months and 12 months post FLASH RT. Owners were recommended to contact the project veterinarians (BB, MLA and KBJ) at any time point if clinical changes occurred between the planned control visits. Due to the COVID-19 pandemic, the assessment was made via phone- or video conference and photographs taken by the owner in a few cases. For each assessment, the tumor was measured using digital calipers, and the skin and mucous membranes were visually inspected. If bone was included in the treatment field, X-rays or computed tomography (CT) were performed at 6- and 12 months post-FLASH RT. When relevant, X-ray or CT was performed at additional time points, for example due to the development of adverse effects. Since the FLASH treatment planning was not based on CT imaging, CT scans were only performed at pre-treatment if relevant for staging purposes.

Response was evaluated using the veterinary RECIST v. 1.0 criteria for solid tumors (14), where complete response (CR) is defined as 100% reduction in the target lesion, partial response (PR) as >30% decrease in the longest diameter (LD) of the target lesion, stable disease (SD) is <30% decrease of the LD and progressive disease (PD) is >20% increase in the LD. Toxicity was evaluated using the VRTOG toxicity grading system (15) for early and late toxicity, which goes from 0 to 3, with 3 being the worst (see **Supplementary Table 1**). Toxicity recorded at the 7 days and 1-month control visits were considered as early toxicity and those recorded at the 3-, 6- and 12 months control visit were considered as late toxicity. Osteoradionecrosis (ORN) was defined as radiographic evidence of bone loss inside the radiation field not related to tumor invasion, or as a clinical observation of non-viable bone tissue. Since there is no validated method for practically assessing salivary production in privately-owned dogs and because clinically significant xerostomia is generally not a problem following conventional RT in dogs (16), salivary flow was not systematically assessed.

### Statistics

Survival was defined as the time in days from FLASH RT to the time of death of any cause. Progression-free survival was defined as the time from FLASH RT until the tumor progressed or the dog died from either tumor or tumor-unrelated causes.

Early data (0-3 months) from dogs no 4 and 9 was included in a previous publication by our group describing feasibility of FLASH RT in canine cancer patients (3).

Data was handled in Google Sheets. Median survival and progression free survival was calculated using GraphPad Prism 9.

## Results

### Canine study population

Eleven dogs were included and treated in the period from September 2020 to November 2021. An overview of patient and treatment parameters can be seen in **Table 1**. The tumor types were malignant melanoma (n=4), squamous cell carcinoma (n=3), soft tissue sarcoma (n=3), and mast cell tumor (n=1). One dog with an extensive malignant melanoma (dog no 3) died of unknown causes two weeks following FLASH RT precluding toxicity and response evaluations beyond the day 7 control visit. One dog with a squamous cell carcinoma (dog no 4) had a relapse of the same tumor type outside the treatment field, which was then treated 6 months following the first site treated. This tumor relapse was considered as a separate tumor in the response evaluations (tumor 4b), making the total number of tumors evaluated for response 11. Another dog with a very extensive malignant melanoma (dog no 11) was retreated 1 month after a partial response to the first treatment. This resulted in nine dogs being treated with a single treatment of FLASH RT and two dogs (dogs no 4 and 11) being treated with two temporally separated treatments.

**Table 1:** Overview of patient and treatment parameters.

	Breed, age, gender	Tumor type	Tumor location	Tumor size (cm) + volume estimate^c^ (cm^3^)	Dose max (Gy)	Bolus (cm)	Field size (cm)^d^
**1**	Basset hound, 11y, ME	Squamous cell carcinoma	Rostral maxillary gingiva	2.4*2.0cm (5cm^3^)	36	0.5	4
**2**	Wirehaired pointer, 11y, ME	Fibrosarcoma	Buccal mucosa	3.2*3.2*2.6 (14cm^3^)	42	–	5
**3**	Dachshund, 11y, MN	Malignant melanoma	Bilateral rostral mandibulary gingiva	>2.5*2 (diffuse) (7cm^3^)	40	0.5	4
**4**	Cross breed, 14y, FE	Basosquamous carcinoma	Intraoral mucosal gingiva	1*2*0.4 (0.4cm^3^)	30	1.5	3
**4b** ^a^	-//-	Basosquamous carcinoma	Extraoral mandibular gingiva	1.5*1.2 (1.1cm^3^)	35	1	3
**5**	Labrador, 7y, FE	Malignant melanoma	Hard palate mucosa	1.8*1.8*0.5 (0.85cm^3^)	35	–	3.5 (tube)
**6**	Galgo espanol, 6y, MN	Papillary squamous cell carcinoma	Rostral mandibular gingiva	1.8*0.5 (0.85cm^3^)	35	0.5	4
**7**	Bernese mountain dog, 7y, ME	Fibrosarcoma	Caudal mandibular gingiva	2.5*1.6*1.0 (2cm^3^)	35	1	3
**8**	Cross breed, 11y, FN	Malignant melanoma	Buccal mucosa	0.9*0.9 (0.4cm^3^)	35	1.5	3
**9**	Cross breed, 10y, FN	Mast cell tumor	Buccal mucosa	0.5*1.0 (diffuse) (0.26cm^3^)	30	1	2
**10**	Dalmatian, 11y ME	Fibrosarcoma	Caudal maxillary gingiva and hard palate	6.0*4.0 (50cm^3^)	30	–	7*5
**11**	Rotteweiler, 9y, FE	Malignant melanoma	Soft palate	>6.0 (113cm^3^)	35 35^b^	- -	5 (tube) 5 (tube)

a, relapse outside RT field in dog no 4. Retreated 6 months after original tumor.

b, second FLASH treatment 4 weeks after the first.

c, estimated by calculating volume for ellipsoid.

d, all fields were circular part from dog no 10.

ME, male entire; MN, male neutered; FE, female entire; FN, female neutered.

None of the dogs were lost to follow-up.

Details on concurrent medications can be found in **Supplementary Table 2**.

### Adverse effects

Detailed information about adverse effects can be found in **Supplementary Table 2**. An overview of the VRTOG toxicity scores per patient and control visit is shown in **Table 2**.

**Table 2:** VRTOG adverse effects.

	Type	Dose (Gy)	Day 7	1 mo	3 mo	6 mo	12 mo	Worst AE	Bone in RT field	ORN
**1**	SCC	36	0	3	1	1	1	3	Yes	No
**2**	FSA	42	0	3	1	3	–	3	Yes	Yes
**3**	OMM	40	0	–	–	–	–	–	Yes	–
**4**	SCC	30	1	0	1	1	–	1	Yes	No
**4b**	SCC	35	1	1	–	–	–	1	–	–
**5**	OMM	35	0	1	3	3	3	3	Yes	Yes
**6**	SCC	35	1	3	1	3	3	3	Yes	Yes
**7**	FSA	35	0	1	1	3	3	3	Yes	Yes
**8**	OMM	35	0	3	1	1	1	3	No	–
**9**	MCT	30	0	1	1	1	1	1	No	–
**10**	FSA	30	0	1	–	–	–	1	Yes	No
**11**	OMM	35 35	0	1	–	–	–	1	No	–

AE, adverse effect; FSA, fibrosarcoma; OMM, oral malignant melanoma; ORN, osteoradionecrosis; MCT, mast cell tumor. RT, radiotherapy; SCC, squamous cell carcinoma.

Overall, six out of ten dogs (60%) living one month or more post FLASH RT developed at least one grade 3 adverse effect. In dogs living >3 months post FLASH RT, six of eight dogs (75%) experienced a grade 3 adverse effect, and in dogs living >6 months, a grade 3 adverse effect had been observed in five of six dogs (83.3%).

At the initial 7-day visit, adverse effects were generally absent, with only two of eleven dogs (18.1%) (dogs no 4 and 6) having a grade 1 adverse effect mainly characterized by mild erythema.

**Figure 1:**
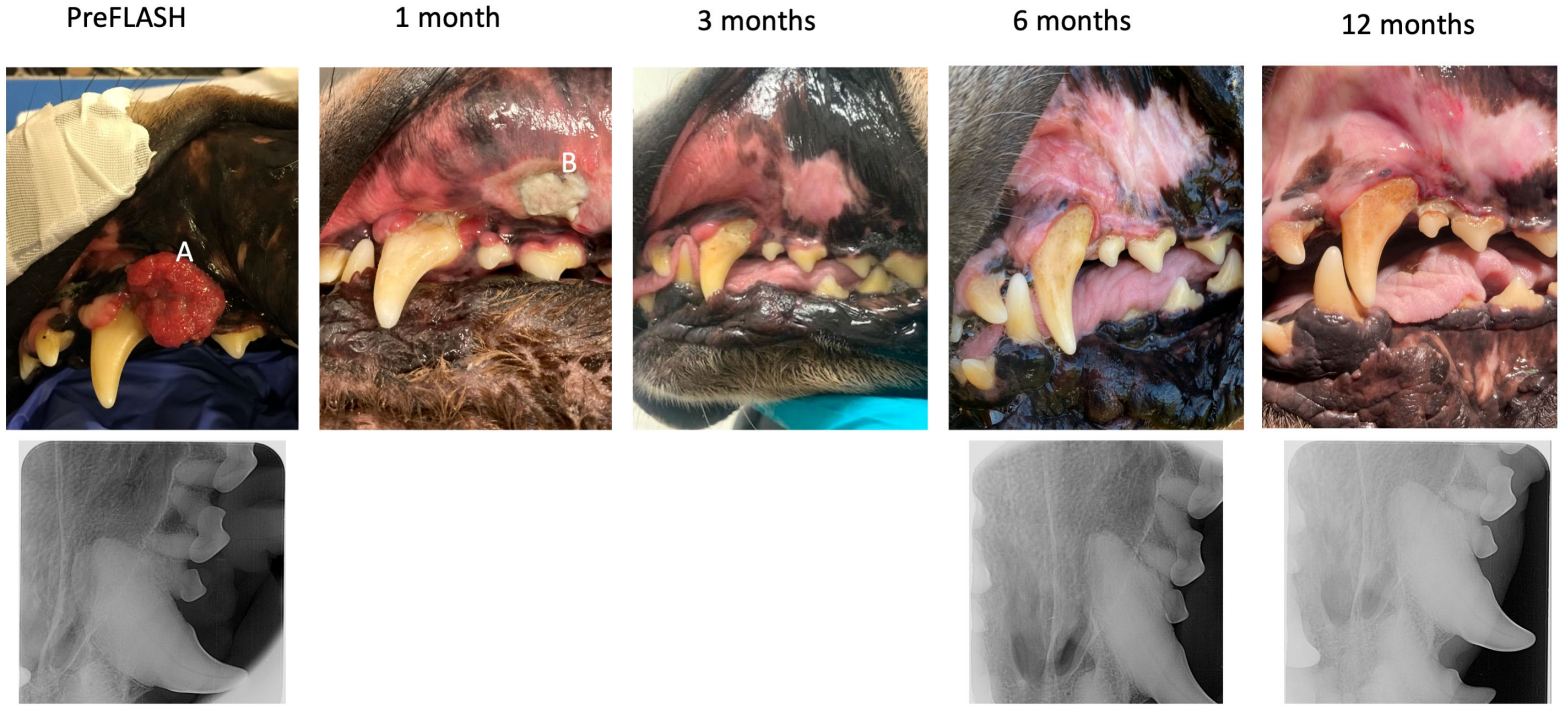
Dog no 1’s treatment response and toxicity, pictures and X-rays. Pictures of dog no 1 from pre-FLASH radiotherapy treatment to 12 months post treatment, incl. X-rays before/after treatment. This dog experienced a complete response and no ORN. **(A)** Squamous cell carcinoma at pre-treatment. **(B)** Grade 3 mucosal ulceration at 1-month post treatment, which subsequently healed.

At the 1-month control visit, adverse effects were either mild grade 1 effects in six of ten dogs (60%), mainly characterized by hyperemia and pigment changes, or grade 3 adverse effects in four of ten dogs (40%). These grade 3 adverse effects were characterized by mucosal ulceration in one dog (dog no 1) (**Figure 1**) or ulceration to underlying haired skin and lip in three dogs (dogs no 2, 6 and 8).

**Figure 2:**
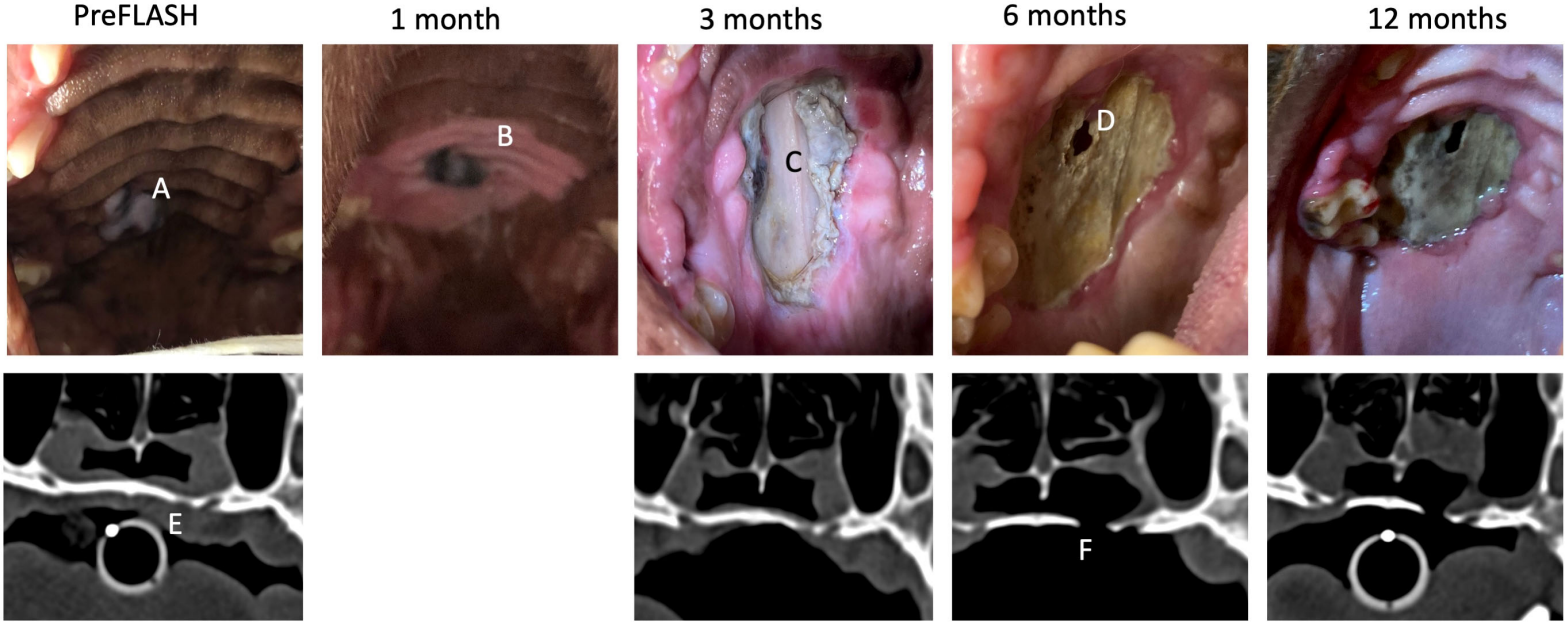
Dog no 5’s treatment response and toxicity, pictures and CT. Pictures of dog no 5 from pre-FLASH radiotherapy treatment to 12 months post treatment, incl. CT before/after treatment. This dog experienced a complete response and ORN. **(A)** Malignant melanoma at pretreatment. **(B)** Delineation of the treatment field by mucosal depigmentation. **(C)** Grade 3 mucosal defect at 3 months post treatment which subsequently progressed. **(D)** ORN and oronasal fistula. **(E)** Decreased density of the palatine bone at pre-treatment at site of later oronasal fistula development. **(F)** Complete defect in palatine bone at 6 months post treatment.

At the 3-month control visit, all these grade 3 ulcerations had healed. In one dog (dog no 5), a grade 3 adverse effect had developed at this point, which was characterized by a necrotic soft tissue defect inside the RT field (**Figure 2**). A CT scan performed at this point in time showed that a minor decrease in palatine bone density present pre-FLASH RT had increased in size and that the rest of the palatine bone was thinning.

**Figure 3:**
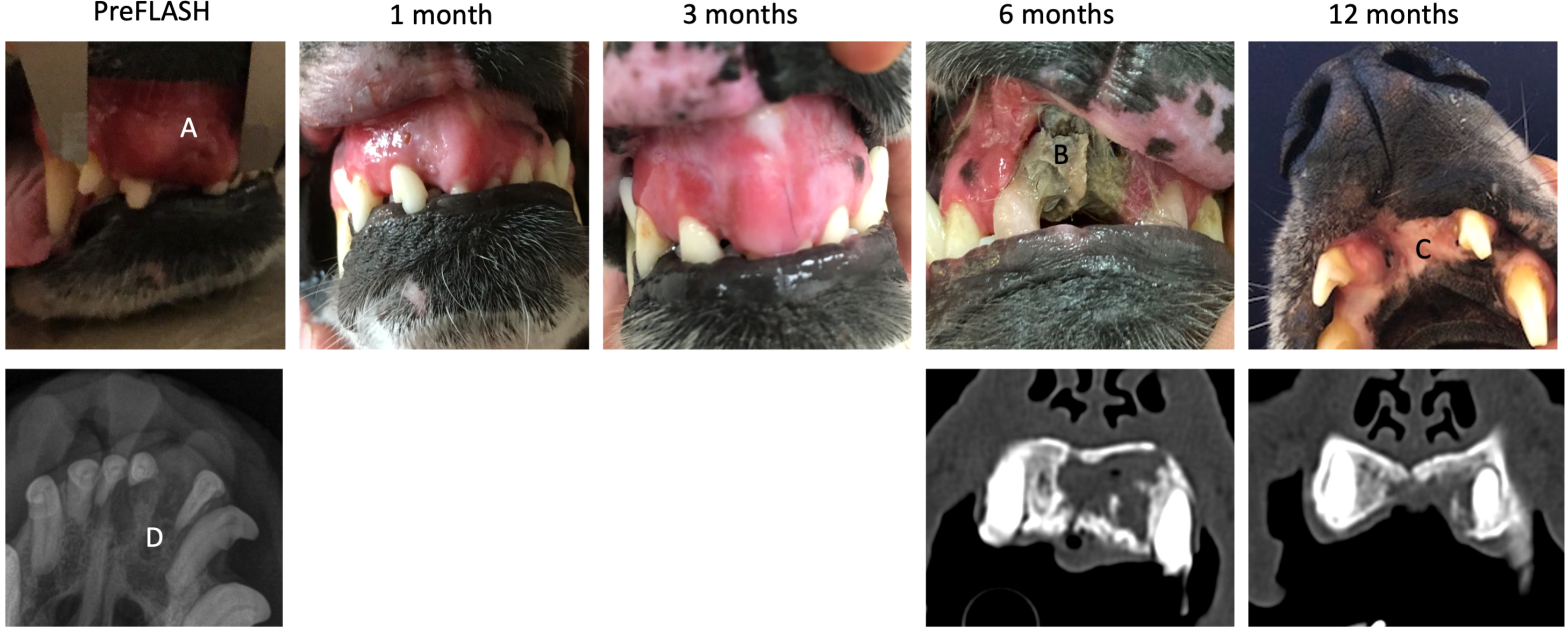
Dog no 6’s treatment response and toxicity, pictures, X-rays and CT. Pictures of dog no 6 from pre-FLASH radiotherapy treatment to 12 months post treatment, incl. X-rays before and CT after treatment. This dog experienced a complete response and ORN. **(A)** Squamous cell carcinoma at pretreatment. **(B)** ORN of maxillary bone at 6 months post treatment. **(C)** Complete coverage of defect by oral mucosa. **(D)** Decreased density of maxillary bone at pre-treatment.

At the 6-month control visit, high grade adverse effects were increasingly common, as four of eight dogs (50%) had a grade 3 adverse effect. The dog with a necrotic soft tissue defect at 3 months (dog no 5) had progressed at 6 months with complete loss of soft tissue inside the RT field and the development of a small oronasal fistula, which could also be appreciated on CT (**Figure 2**). The oronasal fistula appeared to be located at a spot of decreased bone density prior to FLASH RT. Dog no 2 was euthanized at 5 months post FLASH RT due to progressive disease and at euthanasia, a substantial area of mucosal necrosis exposing the underlying necrotic maxillary bone was detected. In dog no 6, a large area of ORN of the rostral maxillary bone was discovered at 6 months post RT. This dog had decreased bone density prior to FLASH RT detected on dental radiography (**Figure 3**). In dog no 7, a small necrotic mucosal defect was detected, which exposed the underlying dental root. CT showed no signs of destruction of the mandibular bone in the area (**Supplementary Figure 3**).

At the 12-month control visit, six dogs were available for evaluation, including two dogs (dogs no 1 and 8) who were euthanized at 11-11.5 months post FLASH, but included in the 12-month evaluations. Three of six dogs (dogs no 5, 6 and 7) had a grade 3 adverse effect characterized by necrotic bone defects. Dog no 7, who had a mucosal defect, but no ORN on CT at the 6-month control visit, now had overt bone necrosis of the underlying mandibular bone detected on CT at the 12-month visit, and the mucosal defect had increased in size (**Supplementary Figure 3**). The ORN and the size of the oronasal fistula appeared to be fairly stable at this time point in dog no 5 (**Figure 2**). In dog no 6, the previously exposed necrotic area had become completely covered by oral mucosa at the 12-month visit, and this dog is currently still alive and doing well at 23 months post-treatment (**Figure 3**).

A total of eight dogs had bone included in the RT field. Six of these were alive at 5-6 months and of these, four (67%) developed ORN during the study period (dogs no 2, 5, 6 and 7). At the 12-month control visit, all four cases with bone in the RT field alive had a CT performed, and 3/4 dogs (75%) had developed ORN. The non-ORN case (dog no 1) was euthanized for an unrelated cause at this time point, so it is unknown if it would have developed ORN later.

Management of the ORN cases is described in **Supplementary Table 2**. The life quality for the dogs having grade 3 adverse effects was continuously monitored and analgesia was supplied as necessary.

Salivary flow was not included in the toxicity evaluations, however no owners mentioned problems with their dogs showing signs of having a dry mouth post treatment.

No dogs were euthanized due to adverse effects in this study.

No additional dogs with oral tumors were enrolled in the study following the first development of a necrotic lesion at 3 months post FLASH RT in dog no 5.

### *In vivo* dosimetry and retrospective dose reconstruction

The average agreement between the prescribed dose and the delivered dose estimated by the *in vivo* film measurements was -0.7% (range -9.4% to 5.4%). For one patient demonstrating ORN (dog no 5), a retrospective reconstruction of the delivered dose distribution was performed based on a pretreatment CT performed for staging purposes. The treatment planning software electronRT (.decimal®, LLC, Sanford, Florida, USA) was used with a beam model of the 10 MeV electron beam of the Elekta Precise linear accelerator. The beam model had previously been validated for calculations in both homogeneous and heterogeneous phantoms using radiochromic film. The dog was treated in the hard palate with an open mouth using a cylindrical PMMA tube, however for the treatment plan, a closed mouth CT scan was used. To reconstruct the treatment setup, the lower jaw was set to the density of air in the CT image. The treatment plan was normalized to the dose measured with radiochromic film at the dose maximum in a solid water phantom using the cylindrical tube. The reconstruction demonstrated a maximum dose outside the target of 42 Gy (120%), with a hotspot in the heterogeneous area (bone and air) posterior to the clinical target volume (CTV) where the ORN developed (bone receiving up to 40 Gy). An illustration of the reconstructed dose distribution in the patient can be found in **Supplementary Figure 4**.

### Efficacy and survival

Detailed information about tumor responses can be found in **Supplementary Table 2**. An overview of the RECIST scoring per patient and control visit is shown in **Table 3**.

**Table 3:** RECIST scoring and survival times.

	Type	Dose	Day 7	1 mo	3 mo	6 mo	12 mo	Best response	PD	Survival time	PFS
**1**	SCC	36	PR	CR	CR	CR	CR	CR	No	345d	345d
**2**	FSA	42	SD	PR	PD	PD	–	PR	Yes	154d	118d
**3**	OMM	40	SD	–	–	–	–	–	–	14d	14d
**4**	SCC	30	SD	CR	CR	CR^a^	–	CR	Yes^a^	281d	152d
**4b** ^a^	SCC	35	SD	PR	CR	–	–	CR	No	–	–
**5**	OMM	35	SD	SD	PR	CR	CR	CR	Yes^a^	488d	488d
**6**	SCC	35	SD	CR	CR	CR	CR	CR	No	Alive at 658d	Alive w/o PD 658d
**7**	FSA	35	SD	SD	PR	CR	CR	CR	No	456d	456d
**8**	OMM	35	SD	PR	CR	CR	CR	CR	No	361d	361d
**9**	MCT	30	SD	CR	CR	CR	PD	CR	Yes	Alive at 991d	378d
**10**	FSA	30	SD	PR	–	–	–	PR	No	53d	53d
**11**	OMM	35 35	PR	PR	–	–	–	PR	Yes	55d	55d

a CR inside RT field, but PD outside RT field.

CR, complete response; FSA, fibrosarcoma; OMM, oral malignant melanoma; MCT, mast cell tumor; PD, progressive disease; PFS, progression-free survival time; PR, partial response; SCC, squamous cell carcinoma; SD, stable disease.

The treatment was generally effective, as eight of eleven tumors had a complete response, and three of eleven had a partial response, giving an overall response rate of 100%.

Nine dogs died during the study period: two due to progressive local disease (dogs no 2 and 11), one due to wide-spread metastases (dog no 5), two due to an uncertain cause, which was possibly tumor-related (dogs no 3 and 10), and four due to unrelated diseases (dogs no 1, 4, 7 and 8). Two of eleven dogs (dogs no 6 and 9) are still alive at the time of data analysis at 658 and 991 days following treatment. Dog no 6 is still in complete remission at 658 days post treatment,

The median progression-free survival was 345 days (range 14-658 days), and the median overall survival was 345 days (range 14-991 days). Survival plots are shown in **Supplementary Figure 2**.

## Discussion

The normal tissue sparing effects of FLASH RT observed in preclinical studies (1, 17) could make FLASH RT especially advantageous for oral cancer patients, who commonly experience serious adverse effects following conventional treatment (11). In the current study, this was investigated in dogs with spontaneous malignant tumors, who were treated with a single fraction of high dose electron FLASH RT.

The treatment was found to be overall feasible and effective. In all tumors available for response evaluation, either a complete response (8/11) or a partial response (3/11) was observed. All four squamous cell carcinomas experienced a durable complete response, which fits well with the expected radiosensitivity of canine oral squamous cell carcinomas (18, 19). Similarly, a durable complete response was observed for a fibrosarcoma, which was beyond what was expected since these tumors are generally only moderately radiosensitive when treated in the macroscopic setting (20). Two of three malignant melanomas also had a long-term complete response, while the last one was too substantial to fit inside the maximum size of our electron treatment field and was treated with a palliative intent. Malignant melanomas are generally radiosensitive to coarse fractionation schemes, which is in alignment with the good response to the single-fraction high dose protocol of this study (21). All in all, the treatment provided, and the group of tumors treated in this study, is too heterogenous to draw any definitive conclusions on FLASH efficacy for canine oral tumors. We did, however, observe tumor responses, which indicates that the single fraction ≥30Gy dose is a clinically effective dose.

The major focus of this study was to evaluate the safety of treating oral malignant tumors with FLASH RT, and, unfortunately, high grade adverse effects were common, even in the early setting. At 1-month post FLASH RT, four cases had a grade 3 adverse effect to the skin or mucosa, which is relatively similar to what one would expect to see after conventionally fractionated RT of oral tumors in dogs, where acute, severe mucositis is a very common observation (22). This contrasted with the sparing effect that we had expected to see in this study based on the limited toxicity observed in our previous dose-escalation trial (3) as well as the multiple preclinical studies showing a general normal-tissue sparing effect of FLASH RT (1, 17, 23). Importantly, the dogs all coped well with the ulcerations, and they had all healed before the 3-month control visit. More worrying was the finding that a single fraction of ≥30 Gy exceeded the tolerance of bone in most cases and resulted in a high risk of high grade late adverse effects in dogs, who had bone included in their RT field. Four of six dogs, who had bone in their treatment field and lived to 5-6 months post treatment, developed ORN. Development of ORN is a well-known risk, when treating human head and neck cancer patients with RT, especially when predisposing factors such as poor dental hygiene or dental extractions are present (11). ORN is generally not a major concern in dogs with oral tumors, partly due to a shorter lifespan post-treatment, but it is estimated that ORN occurs in up to 7.6% of dogs treated with conventionally fractionated RT for an oral tumor (24). That FLASH RT can cause ORN to the structures of the head was also shown in a recent veterinary FLASH RT study where 3/7 cats developed ORN at 9-15 months post treatment (5). The cats were treated with a single fraction of 30 Gy electron FLASH RT to their nasal plane squamous cell carcinomas, and although the treatment appeared to be effective, the investigators had to terminate study inclusion when their first patient presented with progressive bone necrosis at 375 days post treatment. To avoid ORN in human head and neck cancer patients, a dose constraint of 70 Gy to the mandible is generally accepted (25). If the BED of 70 Gy in conventionally fractionated doses (e.g. in 34 fractions) is compared to the BED of 30 Gy delivered in a single fraction, the 30 Gy single-fraction BED is well above (480Gy vs. 142 Gy for an α/β ratio of 2) the dose constraint to the mandible. Importantly, however, it is currently unknown how well the BED model fits with radiotherapy delivered in single fractions and with FLASH RT in general.

The complex nature of the oral cavity means that the prescribed dose was probably not conformally distributed in the tissue. Similar to the observations from the Swiss cat study (5), the reconstructed dose distribution in dog no 5 demonstrated a hot-spot in the heterogeneous area of bone and air posterior to the CTV, which is near the area where the oro-nasal fistula developed. The treatment plan showed up to 120% increase in the planned dose in particular areas and that the bone received up to 40 Gy. These types of issues are common for electron treatments of complex uneven surfaces with air cavities and other types of heterogeneous tissue, and need to be carefully considered for the high doses of single-fraction FLASH RT. We are currently establishing methods for improved treatment planning in order to deliver a more conformal and homogeneous dose to the target (Konradsson et al, accepted for publication in Medical Physics). These methods include CT-based optimized-thickness bolus to prevent hot-spots as well as intensity-modulation of the electron beam to increase the dose homogeneity in the target. Hopefully, this will decrease the occurrence of hot spots in the bone and thereby the risk of ORN development following oral FLASH RT treatments.

Previous data have suggested that optimal normal tissue sparing with FLASH RT is generated when the total dose is delivered in a single pulse or in as short a time as possible (26, 27). The clinical linear accelerator used in this study is limited to a certain pulse structure and dose-per-pulse, and thus the total dose cannot be delivered in a single pulse. Therefore, we cannot exclude that the dose tolerance for FLASH RT would have been higher with a different pulse structure. Although the beam parameters required to observe a FLASH effect are still not clear, the pulse structure of our clinical linear accelerator when operated at ultra-high dose rates has previously been shown to induce a FLASH effect *in vitro* (28, 29) and *in vivo* (Brus et al, in review). In addition, results from our previous phase-I dose-escalation trial including two canine oral tumor patients treated with 30 Gy suggested that FLASH RT would be well-tolerated in the oral cavity. However, these studies have been limited to short-term follow up, and the current study highlights the importance of long-term follow up in FLASH studies before moving forward to human clinical trials.

Apart from conformal treatment planning, the most important tool for decreasing the occurrence of severe late toxicity in RT is treatment fractionation. The knowledge on fractionated FLASH RT, however, is still relatively sparse. A recent paper comparing conventional RT to FLASH RT for whole brain irradiation in mice with glioblastoma, showed that with increasing doses of single-fraction FLASH RT, the mice started to experience neurocognitive deficits (30). When dividing the RT dose into three fractions, long-term tumor control was observed for both modalities, but while conventional RT induced neurocognitive deficits at this scheme, the FLASH RT treated mice showed no deficits compared to non-treated tumor-bearing mice. This study supports the proposition that to fully exploit the potential of FLASH RT, hypo-fractionation is a promising way forward.

This study has several limitations. First, the sample size was small, including dogs with different tumor types, sizes and locations inside the oral cavity. Second, the response assessment was based on imprecise caliper measurements instead of 3D imaging in most cases, which was further complicated by the difficulties in measuring a tumor inside the oral cavity. Third, only six of eleven dogs lived to about a year post treatment, which was the planned follow-up period, and some died very early in the course of the study, meaning that the true risk of for example ORN in this cohort cannot be determined precisely. Finally, the electron FLASH treatments were not based on a treatment planning system, which means that we were unaware of any potential dose hot spots at treatment delivery. Still, we believe that the information regarding toxicity of single-fraction high dose FLASH RT for treating oral tumors that this study provides is highly relevant and important to consider when planning future FLASH RT clinical trials.

FLASH RT holds a promising potential for human cancer patients. With decreased normal tissue toxicity, a hypo-fractionated treatment schedule may become an attractive alternative to standard fractionated therapy, which may cause the patients to be substantially less stressed and inconvenienced by their cancer treatment. Also, a reduced financial burden is expected as patients will need less time off from work and costs related to performing RT may decrease.

Based on the results from the current study, FLASH appears to be effective against canine oral tumors, however with a high risk of development of severe adverse effects. This indicates that fractionation and dose conformity will still be important for FLASH RT to reduce toxicity, when translating this promising treatment modality to the clinic.

## Data availability statement

The original contributions presented in the study are included in the article/**Supplementary Material**. Further inquiries can be directed to the corresponding author.

## Ethics statement

The study was approved by the Local Ethical and Administrative Committee at Department of Veterinary Clinical Sciences, University of Copenhagen, the Danish Experimental Animals Inspectorate (2020–15–0201–00429), the Swedish Board of Agriculture (5.2.18-02830/2020), and the Animal Experiments Committee in Lund, Malmö (5.8.18-14316/2021). The studies were conducted in accordance with the local legislation and institutional requirements. Written informed consent was obtained from the owners for the participation of their animals in this study.

## Author contributions

BB: Conceptualization, Data curation, Formal Analysis, Funding acquisition, Investigation, Methodology, Project administration, Resources, Visualization, Writing – original draft, Writing – review & editing. MA: Conceptualization, Data curation, Investigation, Methodology, Project administration, Resources, Visualization, Writing – review & editing. EK: Conceptualization, Data curation, Formal Analysis, Investigation, Methodology, Project administration, Visualization, Writing – original draft, Writing – review & editing. KJ: Investigation, Resources, Writing – review & editing. SB: Conceptualization, Resources, Writing – review & editing. PM: Conceptualization, Resources, Visualization, Writing – review & editing. CC: Conceptualization, Funding acquisition, Investigation, Methodology, Project administration, Resources, Supervision, Visualization, Writing – review & editing. KP: Conceptualization, Data curation, Formal Analysis, Funding acquisition, Investigation, Methodology, Project administration, Resources, Supervision, Visualization, Writing – review & editing.

## References

[1] Montay-Gruel P Petersson K Jaccard M Boivin G Germond JF Petit B. Irradiation in a flash: Unique sparing of memory in mice after whole brain irradiation with dose rates above 100 Gy/s. Radiotherapy Oncol (2017) 124(3):365–9. doi: 10.1016/j.radonc.2017.05.003 28545957

[2] Vozenin MC De Fornel P Petersson K Favaudon V Jaccard M Germond JF. The advantage of FLASH radiotherapy confirmed in mini-pig and cat-cancer patients. Clin Cancer Res (2019) 25(1):35–42. doi: 10.1158/1078-0432.CCR-17-3375 29875213

[3] Konradsson E Arendt ML Bastholm Jensen K Børresen B Hansen AE Bäck S. Establishment and initial experience of clinical FLASH radiotherapy in canine cancer patients. Front Oncol (2021) 11(May):1–10. doi: 10.3389/fonc.2021.658004 PMC8155542 34055624

[4] Velalopoulou A Karagounis IV Cramer GM Kim MM Skoufos G Goia D. Flash proton radiotherapy spares normal epithelial and mesenchymal tissues while preserving sarcoma response. Cancer Res (2021) 81(18):4808–21. doi: 10.1158/0008-5472.CAN-21-1500 PMC8715480 34321243

[5] Rohrer Bley C Wolf F Gonçalves Jorge P Grilj V Petridis I Petit B. Dose- and volume-limiting late toxicity of FLASH radiotherapy in cats with squamous cell carcinoma of the nasal planum and in mini pigs. Clin Cancer Res (2022) 29:OF1–10. doi: 10.1158/1078-0432.CCR-22-0262 PMC9433962 35421221

[6] Wilson JD Hammond EM Higgins GS Petersson K. Ultra-high dose rate (FLASH) radiotherapy: silver bullet or fool’s gold? Front Oncol (2020) 9(January):1–12. doi: 10.3389/fonc.2019.01563 PMC6979639 32010633

[7] Mascia AE Daugherty EC Zhang Y Lee E Xiao Z Sertorio M. Proton FLASH radiotherapy for the treatment of symptomatic bone metastases. JAMA Oncol (2022) 9(1):62–69. doi: 10.1001/jamaoncol.2022.5843 PMC9589460 36273324

[8] Boss MK. Canine comparative oncology for translational radiation research. Int J Radiat Biol (2022) 98(3):496–505. doi: 10.1080/09553002.2021.1987572 34586958

[9] Paoloni M Khanna C. Translation of new cancer treatments from pet dogs to humans. Nat Rev Cancer (2008) 8(2):147–56. doi: 10.1038/nrc2273 18202698

[10] LeBlanc AK Mazcko CN. Improving human cancer therapy through the evaluation of pet dogs. Nat Rev Cancer. Nat Research; (2020) 20(12):727–42. doi: 10.1038/s41568-020-0297-3 32934365

[11] Sroussi HY Epstein JB Bensadoun RJ Saunders DP Lalla Rv Migliorati CA. Common oral complications of head and neck cancer radiation therapy: mucositis, infections, saliva change, fibrosis, sensory dysfunctions, dental caries, periodontal disease, and osteoradionecrosis. Cancer Med (2017) 6(12):2918–31. doi: 10.1002/cam4.1221 PMC5727249 29071801

[12] Collen EB Mayer MN. Acute oropharyngeal effects of full-course radiation treatment of tumors of the head. Can Vet J (2008) 49(5):509–12. PMC2359501 18512465

[13] Lempart M Blad B Adrian G Bäck S Knöös T Ceberg C. Modifying a clinical linear accelerator for delivery of ultra-high dose rate irradiation. Radiotherapy Oncol (2019) 139:40–5. doi: 10.1016/j.radonc.2019.01.031 30755324

[14] Nguyen SM Thamm DH Vail DM London CA. Response evaluation criteria for solid tumours in dogs (v1.0): A Veterinary Cooperative Oncology Group (VCOG) consensus document. Vet Comp Oncol (2015) 13(3):176–83. doi: 10.1111/vco.12032 23534501

[15] Ladue T Klein MK. TOXICITY CRITERIA OF THE VETERINARY RADIATION THERAPY ONCOLOGY GROUP. Veterinary Radiol Ultrasound (2001) 42(5):475–6. doi: 10.1111/j.1740-8261.2001.tb00973.x 11678573

[16] Gillette EL LaRue SM Gillette SM. Normal tissue tolerance and management of radiation injury. Semin Vet Med Surg Small Anim (1995) 10(3):209–13. 8532978

[17] Favaudon V Caplier L Monceau V Pouzoulet F Sayarath M Fouillade C. Ultrahigh dose-rate FLASH irradiation increases the differential response between normal and tumor tissue in mice. Sci Transl Med (2014) 6(245):245ra93. doi: 10.1126/scitranslmed.3008973 25031268

[18] Grier CK Mayer MN. Radiation therapy of canine nontonsillar squamous cell carcinoma. Can Vet J (2007) 48(11):1189–91. PMC2034434 18050803

[19] Steen F Zandvliet M. Treatment of canine oral papillary squamous cell carcinoma using definitive-intent radiation as a monotherapy—a case series. Vet Comp Oncol (2021) 19(1):152–9. doi: 10.1111/vco.12646 PMC7891416 32975025

[20] Poirier VJ Bley CR Roos M Kaser-Hotz B. Efficacy of radiation therapy for the treatment of macroscopic canine oral soft tissue sarcoma. In Vivo. (2006) 20(3):415–9. 16724681

[21] Blackwood L Dobson JM. Radiotherapy of oral Malignant melanomas in dogs. J Am Vet Med Assoc (1996) 209(1):98–102. 8926220

[22] van der Steen F Zandvliet M. Treatment of canine oral papillary squamous cell carcinoma using definitive-intent radiation as a monotherapy-a case series. Vet Comp Oncol (2021) 19(1):152–9. doi: 10.1111/vco.12646 PMC7891416 32975025

[23] Fouillade C Curras-Alonso S Giuranno L Quelennec E Heinrich S Bonnet-Boissinot S. FLASH irradiation spares lung progenitor cells and limits the incidence of radio-induced senescence. Clin Cancer Res (2020) 26(6):1497–506. doi: 10.1158/1078-0432.CCR-19-1440 31796518

[24] Théon AP Rodriguez C Madewell BR. Analysis of prognostic factors and patterns of failure in dogs with Malignant oral tumors treated with megavoltage irradiation. J Am Vet Med Assoc (1997) 210(6):778–84. 9074679

[25] De Felice F Musio D Tombolini V. Osteoradionecrosis and intensity modulated radiation therapy: An overview. Crit Rev Oncol Hematol (2016) 107:39–43. doi: 10.1016/j.critrevonc.2016.08.017 27823650

[26] Kacem H Almeida A Cherbuin N Vozenin MC. Understanding the FLASH effect to unravel the potential of ultra-high dose rate irradiation. Int J Radiat Biol (2022) 98(3):506–16. doi: 10.1080/09553002.2021.2004328 34788193

[27] Ruan JL Lee C Wouters S Tullis IDC Verslegers M Mysara M. Irradiation at ultra-high (FLASH) dose rates reduces acute normal tissue toxicity in the mouse gastrointestinal system. Int J Radiat OncologyBiologyPhysics (2021) 111(5):1250–61. doi: 10.1016/j.ijrobp.2021.08.004 PMC7612009 34400268

[28] Adrian G Konradsson E Lempart M Bäck S Ceberg C Petersson K. The FLASH effect depends on oxygen concentration. Br J Radiol (2020) 93(1106):20190702. doi: 10.1259/bjr.20190702 PMC7055454 31825653

[29] Adrian G Konradsson E Beyer S Wittrup A Butterworth KT McMahon SJ. Cancer cells can exhibit a sparing FLASH effect at low doses under normoxic *in vitro*-conditions. Front Oncol (2021) 29:11. doi: 10.3389/fonc.2021.686142 PMC8358772 34395253

[30] Montay-Gruel P Acharya MM Gonçalves Jorge P Petit B Petridis IG Fuchs P. Hypofractionated FLASH-RT as an effective treatment against glioblastoma that reduces neurocognitive side effects in mice. Clin Cancer Res (2021) 27(3):775–84. doi: 10.1158/1078-0432.CCR-20-0894 PMC7854480 33060122

