# Supplementary material for: Evaluation of single-fraction high dose FLASH radiotherapy in a cohort of canine oral cancer patients
Source: Front Oncol. 2023 Sep 11;13:1256760. doi: 10.3389/fonc.2023.1256760 (PMC10520273; doi:10.3389/fonc.2023.1256760)
Supplement: Supplementary file 1 [file DataSheet_1.docx]

**Supplementary figures and Tables for CCR publication**

**Supplementary Table 1. Veterinary Radiotherapy Oncology Group (VRTOG) scoring used in this study**

| **Organ/tissue** | **Grade 0** | **Grade 1** | **Grade 2** | **Grade 3** |
| --- | --- | --- | --- | --- |
| **Acute toxicity** | | | | |
| **Skin** | No change over baseline | Erythema, dry desquamation, alopecia/epilation | Patchy moist desquamation without edema | Confluent moist desquamation with edema and/or ulceration, necrosis, hemorrhage |
| **Mucous membranes/oral cavity** | No change over baseline | Injection without mucositis | Patchy mucositis with patient seemingly pain-free | Confluent fibrinous mucositis necessitating analgesia, ulceration, hemorrhage, necrosis |
| **Late toxicity** | | | | |
| **Skin/hair** | None | Alopecia, hyperpigmentation, leukotrichia | Asymptomatic induration (fibrosis) | Severe induration causing physical impairment, necrosis |
| **Bone** | None | Pain on palpation | Radiographic changes | Necrosis |

Modified from: Ladue, T., & Klein, M. K. (2001). TOXICITY CRITERIA OF THE VETERINARY RADIATION THERAPY ONCOLOGY GROUP. In *Veterinary Radiology & Ultrasound, Vnl* (Vol. 42, Issue 5).

**Supplementary Table 2. Detailed patient, response and toxicity information.**

|  | **Treatment** | **Day 7** | **Day 28** | **3 mo** | **6 mo** | **12 mo** | **Other notes** |
| --- | --- | --- | --- | --- | --- | --- | --- |
| **1** | Tumor 2.4*2.0  XR shows no signs of bony lysis.  Bone and teeth in RT field. | Slight mucosal erythema. Tumor 1 cm Ø. | Necrotic ulcer in cheek in RT field, no observable tumor. | Scar at previous ulcer location, no observable tumor | Scar at previous ulcer location, XR shows no bony lysis. No observable tumor. | 11.3 months post FLASH. Scar at previous ulcer location, XR shows no bony lysis. No observable tumor. Euth. | Concurrent medications: methylprednisolone (atopy) and pregabalin (orthopedic pain). Euth at 11.3months post RT for unrelated cause, still in CR at this point. |
| **2** | Tumor 3.2*3.2*2.6 cm, necrotic cavity in tumor.  Bone and teeth in RT field. | Still necrotic cavity, tumor 3.3 cm Ø | Haired skin deep to RT field ulcerated with moist desquamation. No mucosal changes. Tumor subjectively decreased in size^a.^ | Alopecia, tumor markedly increased in size^a.^ | 5 mo post FLASH. Alopecia, mucosal necrosis exposing bone adjacent to tumor. Tumor increased in size^a^. Euth. | - | Concurrent medications: meloxicam.  Euth due to PD at 5mo post FLASH. Autopsy confirms bone necrosis in RT field. |
| **3** | Tumor >2.5*2.0 cm (diffuse). XR shows no signs of bony lysis.  Bone and teeth in RT field. | No mucosal changes, no change in tumor size^a^ | - | - | - | - | Concurrent medications: meloxicam. Dog dies acutely in owners’ home 14 days post FLASH, unknown cause, but dog has multiple health problems |
| **4** | Tumor 1.0*2.0*0.4 cm, XR shows decreased bone density.  Bone and teeth in RT field. | Mild erythema, tumor subjectively decreased in size^a^ | Normal mucosa, no observable tumor. | Depigmented mucosa, no observable tumor. | Depigmented mucosa, no signs of tumor in RT field, but PD outside RT field. XR shows decreased bone density comparable to pretreatment. | - | Concurrent medications: robenacoxib. No signs of tumor regrowth inside RT field at euth 9 mo post FLASH (for unrelated mammary tumor). |
| **4b** | Regrowth outside RT field. Tumor 1.6*1.2 cm. XR shows decreased bone density comparable to pretreatment of tumor 4.  Bone and teeth in RT field. | Superficial gingival cavity in RT field at site of tumor, no adverse effects, tumor:  1.1*1.1*0.5 cm. | Gingival cavity decreased in size, no adverse effects, tumor: 1.0*0.8*0.4 cm. | Normal gingiva, no observable tumor. Euth. | - | - | See no 4. |
| **5** | Tumor 1.8*1.8*0.5 cm, CT shows small 1.6 mm punctate lucency in palatine bone.  Bone but no teeth in RT field. | Normal mucosa, no change in tumor size^a^ | Depigmentation in RT field, tumor subjectively smaller^a^ | Tissue in RT field appears necrotic.  CT shows increased size of punctate lucency in palatine bone (2.9 mm) and general thinning of palatine bone in RT field. Tumor subjectively smaller^a^ | Large necrotic defect in tissue in entire RT field including a small oro-nasal fistula. CT shows a palatine bone defect of 4*8.8mm. No observable tumor. | Large necrotic defect in tissue in entire RT field including a small oro-nasal fistula. CT shows a palatine bone defect of 3*12.6 mm. No observable tumor. | Concurrent medications: methylprednisolone (atopy). ORN treated with pregabalin and paracetamol + antibiotics. Euthanized 16mo post treatment due to progressive pulmonary metastasis. Still in CR inside RT field, but PD is detected outside the dental arcade. |
| **6** | Tumor 1.8*0.5 cm. XR shows local bony destruction.  Bone and teeth in RT field. | Lip erythema, tumor subjectively decreased in size^a^ | Ulceration lip, depigmentation, no observable tumor | Depigmentation lip, no observable tumor | Large necrotic defect of bone in RT field, mild atrophy of lip, CT shows irregular destruction of the rostral maxillary bone. No observable tumor. | Necrotic defect has been resorbed and the defect closed by oral mucosa. Lip partly atrophied exposing teeth. No observable tumor. | Concurrent medications: meloxicam. ORN treated with pregabalin, vitamin E and pentoxifylline + antibiotics. Alive and in CR at time of data analysis. |
| **7** | Tumor 2.5*1.6*1.0 cm.  CT shows no signs of bony lysis.  Bone and teeth in RT field. | Normal mucosa, tumor: 2.4*1.8*1.0cm. | Slight hyperemia, tumor: 1.9*1.3*0.8cm. | Depigmented mucosa, tumor 1.0*0.5cm | Small necrotic tissue defect exposing dental root in RT field. CT shows no signs of bony lysis. No observable tumor. | Necrotic tissue defect exposing dental root. CT shows complete bone lysis around affected tooth. No observable tumor. | Concurrent medications: meloxicam initially, later firocoxib. No additional treatment for ORN. Euth at 14 mo post FLASH due to a general decrease in health, still in CR at this point. |
| **8** | Tumor 0.9*0.9 cm.  No bone or teeth in RT field. | Normal mucosa, tumor: 8mm | Normal mucosa, but haired skin under RT field is ulcerated, tumor further decreased in size | Depigmented mucosa, ulcerated skin has healed, no observable tumor | Depigmentation skin and mucosa, no observable tumor | 11 mo post FLASH. Depigmented scar tissue in mucosa, alopecia and depigmentation in skin, no observable tumor. Euth. | Concurrent medications: trilostane for hyperadrenocorticism.  Euth for unrelated problem 11mo post FLASH, still in CR at euth. |
| **9** | Tumor 0.5*1.0 cm (diffuse). No bone or teeth in RT field. | Normal mucosa, no change in tumor size^a^ | Alopecia and hyperpigmentation to skin, no observable tumor. | Alopecia, no observable tumor. | Alopecia, pigment change, no observable tumor. | Alopecia, relapse of tumor in RT field^c^. | Concurrent medications: vinblastine/prednisolone. Relapse at 11mo. Alive at time of data analysis. |
| **10** | Tumor 6.0*4.0 cm, heterogenes.  Bone and teeth in RT field. | Normal mucosa, tumor subjectively decreased in size^a.^ | Hypopigmented mucosa, tumor hardly discernible | - | - | - | Concurrent medications: firocoxib. Euthanized 2mo post FLASH due to breathing difficulties. |
| **11** | Tumor >6.0 cm Ø, diffuse.  Bone and teeth in RT field. | Normal mucosa, tumor subjectively decreased in size^a^ and dogs has less difficulty breathing. | Depigmentation, subjective decrease in tumor size, marked clinical improvement. Retreated^b^. | Progressive disease | - | - | Concurrent medications: firocoxib. Full tumor not included in RT field due to large size and location, treated with palliative intent. Retreated 4 weeks following the first FLASH dose. Euth at 2mo post FLASH due to PD. |

a. Tumor cannot be accurately measured on awake dog. b: Retreated 1 month after first FLASH fraction. c: Relapse confirmed by cytology.

CR: complete response. CT: computed tomography. Euth: euthanasia. ORN: osteoradionecrosis. PD: progressive diseased. RT: radiotherapy. XR: X-rays.

**Supplementary Figure 1. Setup with and without PMMA applicator**

**
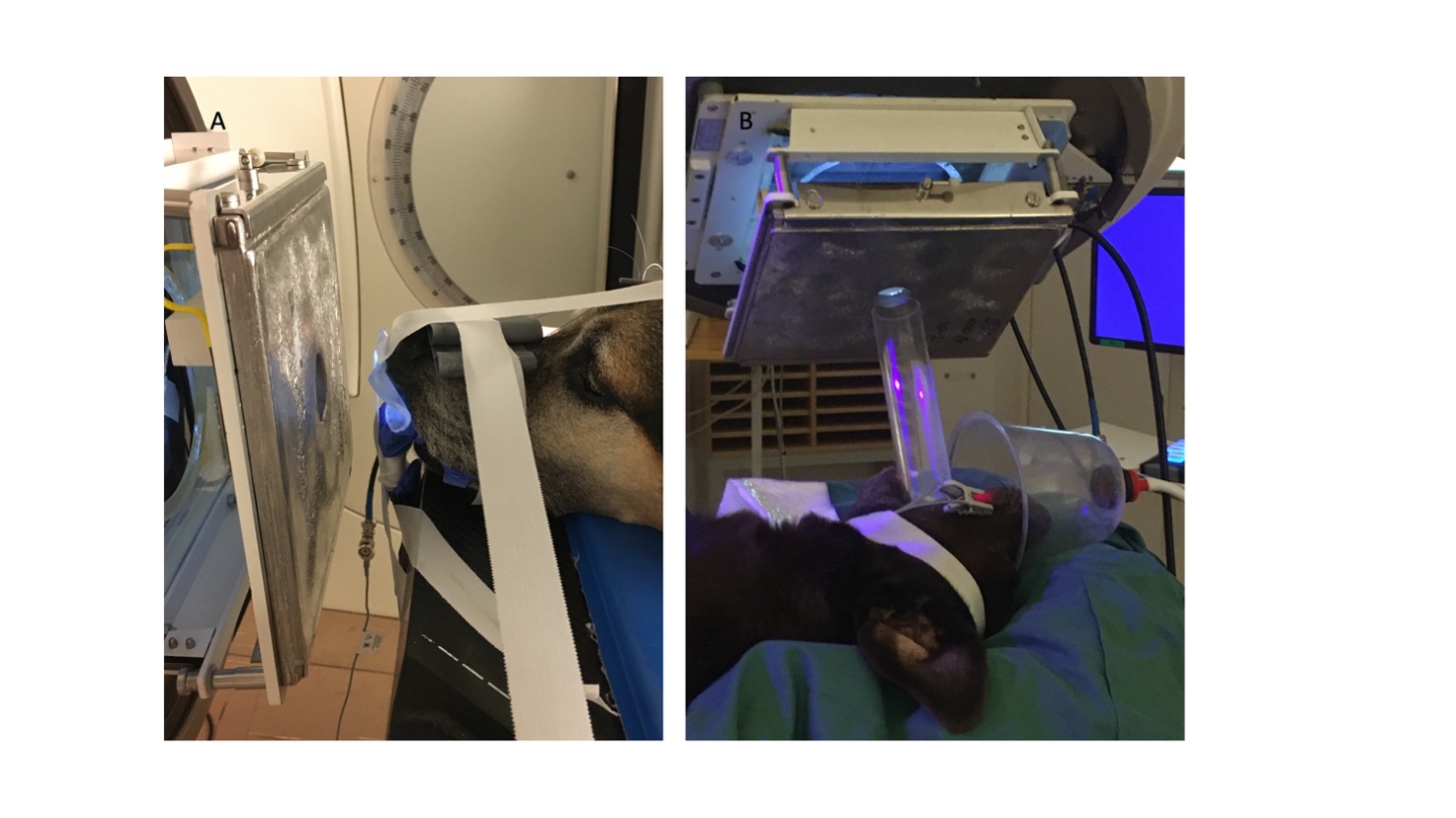
**

A: Setup without PMMA applicator. Dog no 6 with squamous cell carcinoma of the maxillary gingiva. B: Setup with PMMA applicator. Dog no 5 with malignant melanoma of the palate.

**Supplementary Figure 2. Progression-free survival and overall survival plots**

**
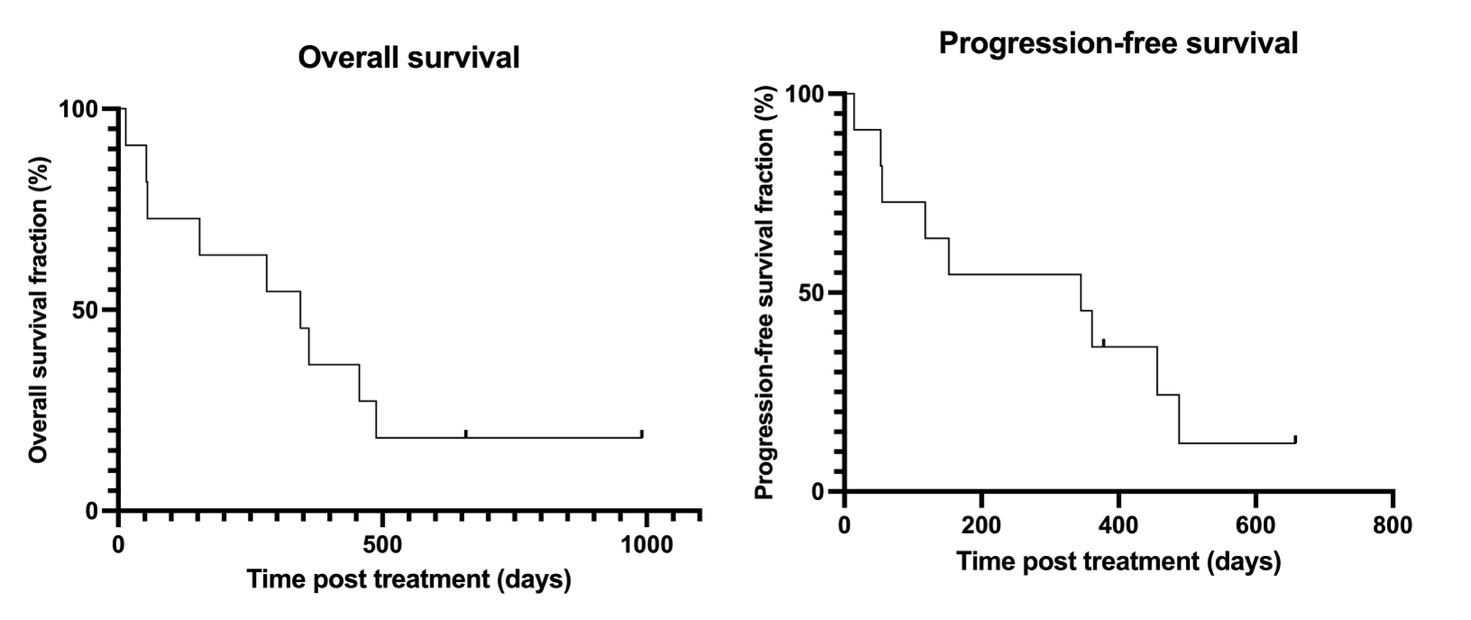
**

Left: Kaplan-Meier plot displaying the overall survival. Right: Kaplan-Meier plot displaying the progression-free survival. Vertical bars represent censored data.

**Supplementary Figure 3. Dog no 7 treatment response and toxicity, pictures and CT**

**
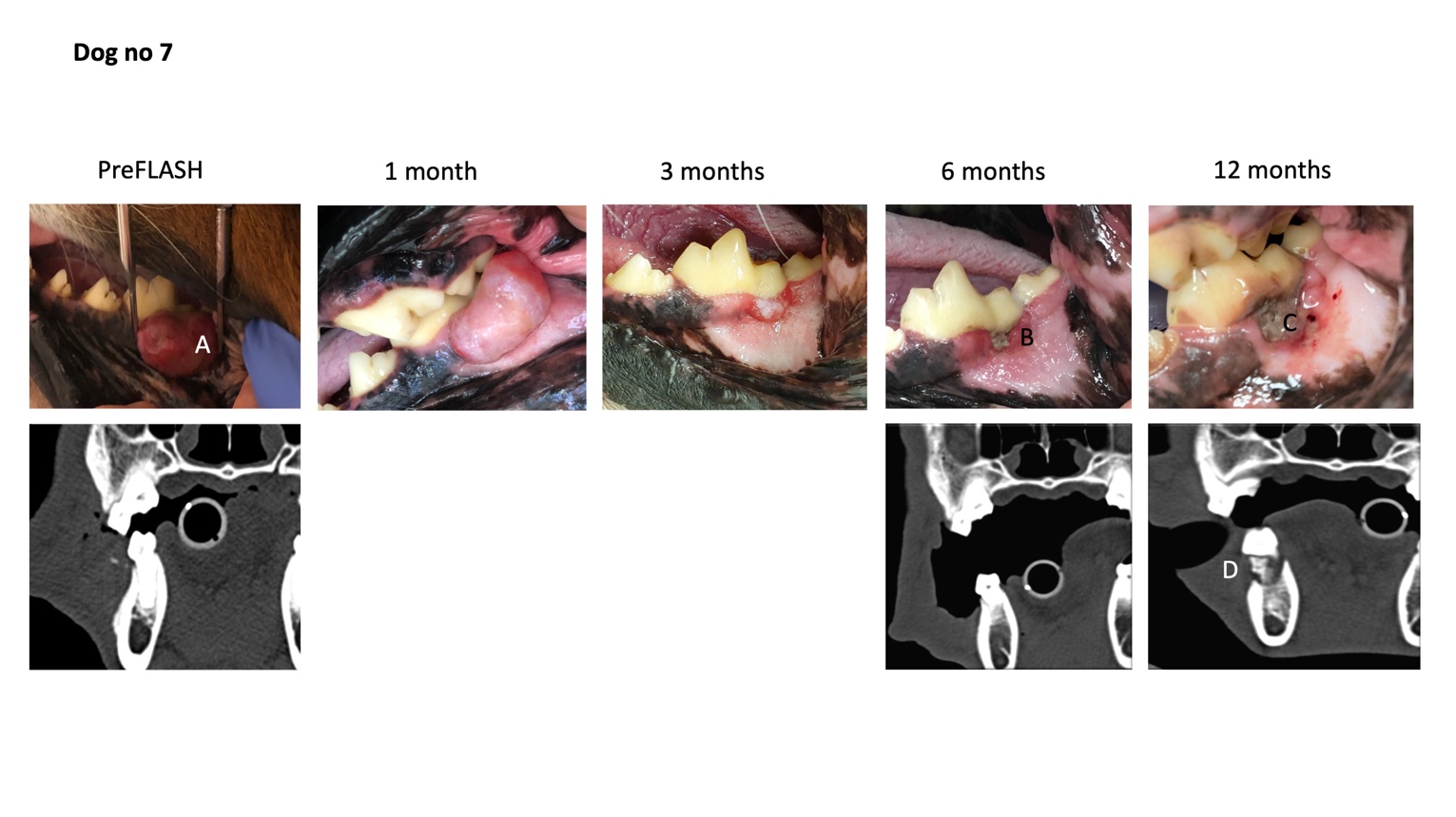
**

Pictures of dog no 7 from preFLASH radiotherapy treatment to 12 months post treatment incl. CT before/after treatment. This dog experienced a complete response and ORN. A: Fibrosarcoma at pretreatment. B: Grade 3 gingival defect at 6 months post treatment which subsequently progressed. C: Progression of gingival defect. D: ORN of mandibular bone at 12 months post treatment.

**Supplementary Figure 4. Reconstructed dose distribution of dog no 5 created in the treatment planning software electronRT**

**
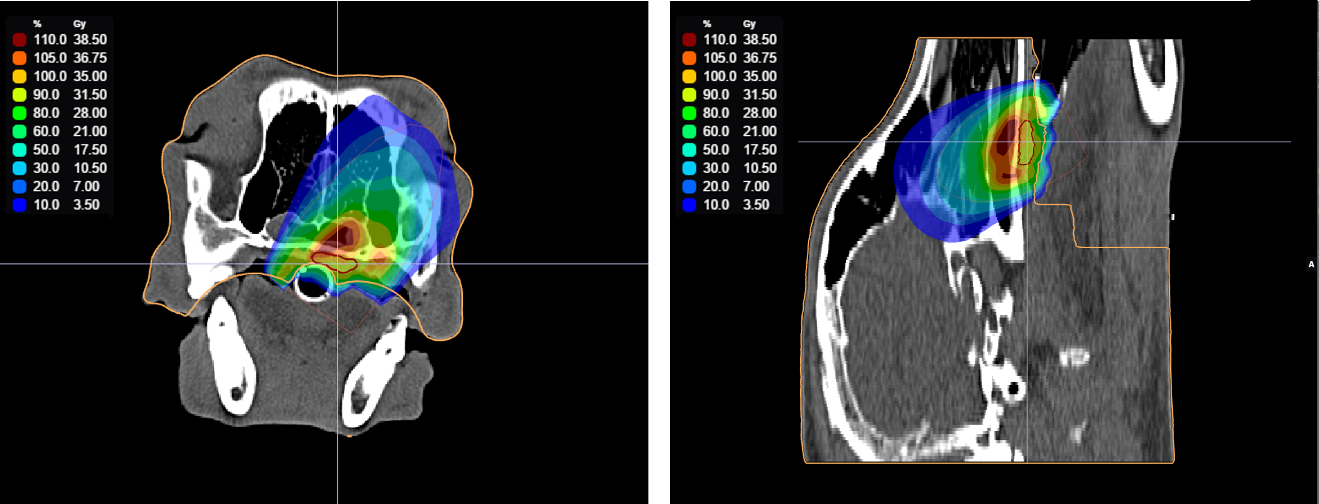
**

Transverse (left panel) and sagittal (right panel) view of CT slices overlayed with the reconstructed dose distribution. The CTV is delineated by a red contour. The prescribed dose was 35Gy to the CTV, and the relative/absolute dose levels are illustrated by color shades. The treatment plan revealed a maximum dose outside the target of 42Gy (120%), and a hot spot in the heterogeneous area posterior to the CTV with the bone receiving up to 40Gy (114%).
